# Novel quantitative immunohistochemical analysis for evaluating PD-L1 expression with phosphor-integrated dots for predicting the efficacy of patients with cancer treated with immune checkpoint inhibitors

**DOI:** 10.3389/fimmu.2023.1260492

**Published:** 2023-09-18

**Authors:** Ryotaro Ohkuma, Sakiko Miura, Satoshi Muto, Yoshitaka Toyomasu, Yuki Fujimoto, Katsuaki Ieguchi, Nobuyuki Onishi, Takashi Shimizu, Makoto Watanabe, Daisuke Takayanagi, Tsubasa Goshima, Atsushi Horiike, Kazuyuki Hamada, Hirotsugu Ariizumi, Masahiro Shimokawa, Yuya Hirasawa, Tomoyuki Ishiguro, Risako Suzuki, Nana Iriguchi, Toshiaki Tsurui, Emiko Mura, Sachiko Takenoshita, Kazuki Numajiri, Naoyuki Okabe, Kiyoshi Yoshimura, Mayumi Tsuji, Yuji Kiuchi, Toshiki Yajima, Hideyuki Ishida, Hiroyuki Suzuki, Toshiko Yamochi, Shinichi Kobayashi, Takuya Tsunoda, Satoshi Wada

**Affiliations:** ^1^ Division of Medical Oncology, Department of Medicine, School of Medicine, Showa University, Tokyo, Japan; ^2^ Department of Clinical Diagnostic Oncology, Clinical Research Institute for Clinical Pharmacology and Therapeutics, Showa University, Tokyo, Japan; ^3^ Department of Pathology, Showa University School of Medicine, Tokyo, Japan; ^4^ Department of Chest Surgery, School of Medicine, Fukushima Medical University, Fukushima, Japan; ^5^ Department of Digestive Tract and General Surgery, Saitama Medical Center, Saitama Medical University, Saitama, Japan; ^6^ Clinical Research Institute for Clinical Pharmacology and Therapeutics, Showa University, Tokyo, Japan; ^7^ Department of Pharmacology, School of Medicine, Showa University, Tokyo, Japan; ^8^ Pharmacological Research Center, Showa University, Tokyo, Japan; ^9^ Department of General Surgical Science, Graduate School of Medicine, Gunma University, Gunma, Japan; ^10^ Department of Clinical Immuno Oncology, Clinical Research Institute for Clinical Pharmacology and Therapeutics, Showa University, Tokyo, Japan; ^11^ Department of General Thoracic Surgery, Faculty of Medicine, Kagawa University, Kagawa, Japan

**Keywords:** phosphor-integrated dots, fluorescent nanoparticles, immunohistochemistry, imaging pathology, quantitative evaluation, PD-L1, immune-checkpoint inhibitors, biomarker

## Abstract

**Introduction:**

Programmed cell death ligand 1 (PD-L1) expression in tumor tissues is measured as a predictor of the therapeutic efficacy of immune checkpoint inhibitors (ICIs) in many cancer types. PD-L1 expression is evaluated by immunohistochemical staining using 3,3´-diaminobenzidine (DAB) chronogenesis (IHC-DAB); however, quantitative and reproducibility issues remain. We focused on a highly sensitive quantitative immunohistochemical method using phosphor-integrated dots (PIDs), which are fluorescent nanoparticles, and evaluated PD-L1 expression between the PID method and conventional DAB method.

**Methods:**

In total, 155 patients with metastatic or recurrent cancer treated with ICIs were enrolled from four university hospitals. Tumor tissue specimens collected before treatment were subjected to immunohistochemical staining with both the PID and conventional DAB methods to evaluate PD-L1 protein expression.

**Results:**

PD-L1 expression assessed using the PID and DAB methods was positively correlated. We quantified PD-L1 expression using the PID method and calculated PD-L1 PID scores. The PID score was significantly higher in the responder group than in the non-responder group. Survival analysis demonstrated that PD-L1 expression evaluated using the IHC-DAB method was not associated with progression-free survival (PFS) or overall survival (OS). Yet, PFS and OS were strikingly prolonged in the high PD-L1 PID score group.

**Conclusion:**

Quantification of PD-L1 expression as a PID score was more effective in predicting the treatment efficacy and prognosis of patients with cancer treated with ICIs. The quantitative evaluation of PD-L1 expression using the PID method is a novel strategy for protein detection. It is highly significant that the PID method was able to identify a group of patients with a favorable prognosis who could not be identified by the conventional DAB method.

## 1 Introduction

Immune checkpoint inhibitors (ICIs) have been developed as antitumor agents with mechanisms completely different from those of conventional cytotoxic chemotherapies for patients with cancer. Immune checkpoint mechanisms were originally intended to regulate excessive autoimmune responses. However, in the cancer microenvironment, cancer cells use immune checkpoints to escape antitumor immune responses, involving pathways mediated by immune checkpoint molecules such as programmed death protein-1 (PD-1), cytotoxic T-lymphocyte-associated protein 4 (CTLA-4), and various other factors. PD-1 and its ligand programmed cell death ligand 1 (PD-L1) are fundamental factors in the immune checkpoints that interfere with immune escape (1). The clinical efficacy and safety profile of anti-PD-1 and anti-PD-L1 antibodies have been demonstrated in various cancer types (2). CTLA-4 negatively regulates immune function through its interaction with B7 (CD80/CD86) expressed on the surface of cancer cells, and its competitive action with CD28, which activates T cells (3, 4). Anti-CTLA-4 antibodies have shown efficacy in multiple types of cancers as monotherapy or in combination with other ICIs, especially the anti-PD-1 antibody. The therapeutic effects of ICIs have had a strong impact on cancer treatment, not only by improving response rates and prolonging progression-free survival (PFS) but also by providing a “long-tail effect,” which is characterized by the long-term overall survival (OS) of patients with cancer. Thus, ICIs have become a significant breakthrough in cancer immunotherapy, showing remarkable efficacy against various cancer types by suppressing checkpoint-mediated immune escape (5). **Table 1** summarizes the results of representative phase III pivotal studies that evaluated the efficacy of ICI treatment and served as the basis for approval (6–14). In contrast, many clinical trials have reported that ICIs are ineffective in all patients with cancer, especially ICI monotherapy, with an efficacy rate of only 10–30% (15). Therefore, further improvement in the efficacy of ICIs is necessary. The expression of PD-L1 molecules, high-frequency microsatellite instability, and tumor mutation burden have been identified as potential predictive biomarkers of the therapeutic response to ICIs; however, no definitive factors have been reported to correctly predict the treatment response to ICIs (16). Therefore, superior predictive biomarkers with high therapeutic efficacy and prognostic value are urgently needed.

**Table 1:** Main results of the previous pivotal phase III trials of immune checkpoint inhibitors in advanced cancer.

		Trial	Phase	Follow-up duration	Cancer type	Regimen	Treatment line	PD-L1 expression	Median PFS, months (95% CI)	Median OS, months (95% CI)	Reference no.
Monotherapy	1	KEYNOTE-024	Phase III	5y	NSCLC	Pembrolizumab, 200 mg q3w	1st	TPS>50%	7.7 (6.1-10.2)	26.3 (18.3-40.4)	(6)
	2	CheckMate 057	Phase III	5y	Non-squamous NSCLC	Nivolumab, 3 mg/kg q2w	2nd, 3rd	All comers	2.5 (2.2-3.5)	11.1 (9.2-13.1)	(7)
	3	OAK	Phase III	–	NSCLC	Atezolizumab, 1200 mg q3w	2nd, 3rd	All comers	2.8 (2.6-3.0)	13.8 (11.8-15.7)	(8)
	4	ATTRACTION-2	Phase III	3y	Gastric cancer (adenocarcinoma)	Nivolumab, 3 mg/kg q2w	3rd~	All comers	1.6 (1.5-2.3)	5.3 (4.6-6.4)	(9)
	5	KEYNOTE-045	Phase III	2y	Urothelial cancer	Pembrolizumab, 200 mg q3w	2nd~	All comers	2.1 (1.9-2.1)	10.1 (8.0-12.3)	(10)
	6	CheckMate 141	Phase III	2y	Head and neck (Squamous carcinoma)	Nivolumab, 240 mg q2w	2nd~	All comers	2.1 (1.9-3.2)	7.7 (3.1-12.6)	(11)
	7	CheckMate 067	Phase III	6.5y	Malignant melanoma	Nivolumab, 3 mg/kg q2w	1st	All comers	6.9 (5.1-10.2)	36.9 (28.2-NR)	(12)
Combination therapy	8	CheckMate 067	Phase III	6.5y	Malignant melanoma	Nivolumab, 1mg/kg q3w + ipilimumab, 3mg/kg q3w	1st	All comers	11.5 (8.7-19.3)	72.1 (38.2-NR)	(12)
	9	KEYNOTE-189	Phase III	>2y	Non-squamous NSCLC	Pembrolizumab, 200 mg q3w + platinum doublet therapy, q3w	1st	All comers	9.0 (8.1-10.4)	22.0 (19.5-24.5)	(13)
	10	Impower 130	Phase III	–	Non-squamous NSCLC	Atezolizumab, 1200 mg q3w + CBDCA(q3w) + nab-PTX(q1w)	1st	All comers	7.0 (6.2-7.3)	18.6 (16.0-21.2)	(14)

NSCLC, non-small cell lung cancer; PD-L1, programmed cell death ligand 1; PFS, progression-free survival; OS, overall survival; TPS, Tumor Proportion Score; CI, confidence interval; no., number; q1w, once weekly; q2w, once every 2 weeks; q3w, every 3 weeks.

To date, most studies on the biomarkers of ICI treatment have focused on the analysis of PD-L1 expression in tumor tissues using immunohistochemistry (IHC). PD-L1 expression in tumor tissue has been used as a biomarker in determining cancer treatment with ICIs (17), but is not used universally in many types of cancers. PD-L1 expression detected by IHC analysis has several limitations as a predictive biomarker. Although treatment responses to anti-PD-1 or anti-PD-L1 antibody therapies are associated with the expression of PD-L1 protein in tumor tissues, approximately 10–40% of PD-L1-negative patients also respond to anti-PD-1 or anti-PD-L1 therapies (18, 19). Conversely, we often encounter cases where PD-L1-positive patients do not respond to ICIs. This contradiction is considered to be caused by PD-L1 expression as determined by IHC and visual inspection by pathologists, which limits the objectivity of determining PD-L1 expression levels. In other words, the evaluation of PD-L1 expression performed by pathologists using IHC is limited because it does not provide a quantitative evaluation and lacks objectivity. Another limitation of the IHC method is that the immunohistochemical staining method of the PD-L1 molecule is based on the intensity of the color visualized by the chromogenic agent 3,3´-diaminobenzidine (DAB). In the conventional IHC method generally used in the clinical setting, tissue sections are incubated with primary antibodies and biotin-labeled secondary antibodies, followed by a reaction with streptavidin-labeled horseradish peroxidase (HRP) and a secondary antibody, and then with HRP and DAB chromogen. Therefore, in IHC-DAB, the staining intensity depends on the enzymatic activity of HRP and is greatly affected by the air temperature, reaction time, and HRP substrate concentration (20). Consequently, the quantitative sensitivity and dynamic range of conventional IHC methods using DAB for pathological diagnosis are poor.

**Figure 1:**
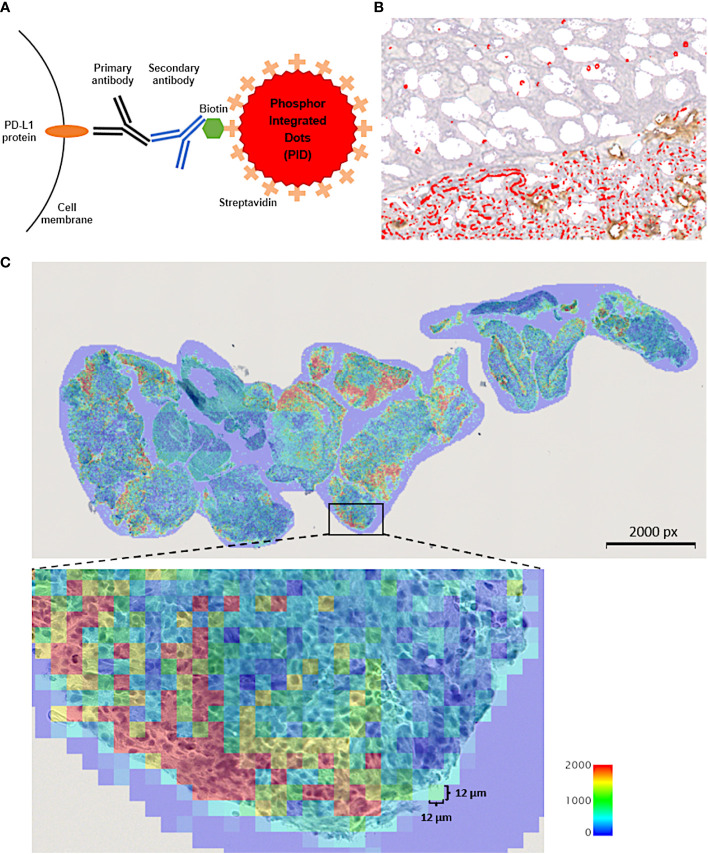
**(A)** Schematic explanation for the phosphor-integrated dot (PID) imaging of cancer tissues. The target protein, programmed cell death ligand 1 (PD-L1) molecules in this study, in tumor tissue were immunostained with monomeric and biotinylated monoclonal primary and monoclonal secondary antibodies. Then, the samples were stained with streptavidin-coated PID by biotin-streptavidin binding. **(B)** Immunohistochemistry of cancer tissue using PID staining. Red spots on the tumor cells indicate PID particles. **(C)** The number of PID particles were quantified in whole regions of tumor tissue specimen. The number of PD-L1-positive PID particles per 12 µm ×12 µm in the tumor cell nuclei were counted and shown as a heat map. The “PD-L1 PID score” for each case was calculated as the mean value of the number of PID particles per 12 µm × 12 µm area within each tissue specimen. px, pixel.

As described above, the scoring method of the former IHC is dependent on the staining intensity, so it is not completely quantifiable. To overcome these limitations of IHC-DAB, we focused on the phosphor-integrated dot (PID) method using fluorescent nanoparticles, a novel protein quantification method developed by Konica Minolta, Inc. (Tokyo, Japan). Although existing IHC-DAB coloration systems have quantitative problems in low-expression groups, the PID system has a wide dynamic range, enabling the detection of both low- and high-expression groups (21). Fluorescent IHC can effectively improve the quantitative sensitivity of conventional IHC-DAB; however, tissue autofluorescence hinders sensitivity (22). To improve this fluorescent IHC autofluorescence deficiency, the PID method is further characterized by the 100-fold luminance of conventional fluorescent nanoparticles and high lightfastness, which is >10 times higher than those of existing fluorescent dyes (21). Given these characteristics, the system is expected to measure protein expression more quantitatively, including in a range undetectable by existing IHC. Compared to conventional IHC-DAB, the PID method provides more objective data on protein expression because it is possible to count the number of PID particles that bind in a one-to-one fashion with antibodies in each cell. Additionally, an image processing method was developed to calculate the PID particle counts for the acquired images. We compared the characteristics of the PID schemas with those of conventional IHC. We present a schema outlining the PID method (**Figure 1A**) and a table comparing the features of each method (**Table 2**). Recent studies have explored the application of fluorescent nanoparticles in quantitative diagnostics because of their high photostability and brightness; however, their clinical application has not yet been achieved. Although two previous studies evaluated PD-L1 expression using the PID method (23, 24), it is unclear whether it can be a predictive biomarker for the therapeutic efficacy of ICIs, such as anti-PD-1, anti-PD-L1, or anti-CTLA-4 antibodies.

**Table 2:** Methodology for quantifying protein expression.

Method	Advantage	Disadvantage
**FACS**	Suitable for measuring the total amount of protein present in the cell.	Not possible to evaluate both cell morphology and protein expression-dependent characteristics simultaneously.
**IHC**	Both cell morphology and protein expression-dependent characteristics can be evaluated simultaneously.	The intensity of DAB staining depends on the enzymatic activity of HRP and is greatly affected by reaction time, temperature, and HRP substrate concentration; thus, the quantitative sensitivity of IHC-DAB is low.
**Fluorescent IHC**	Effectively increases the quantitative sensitivity of conventional IHC.	Poor photostability and interference with tissue autofluorescence.
**IHC with PIDs**	High fluorescence intensity and high photostability. Newly developed image processing method enables calculation and quantification of the number of PID particles in the obtained images.	Requires specific equipment for PID analysis.

FACS, fluorescence-activated cell sorting; IHC, immunohistochemistry; PIDs, phosphor-integrated dots; DAB, 3,3´-diaminobenzidine; HRP, horseradish peroxidase.

Application of the PID method is expected to overcome the limitations of IHC-DAB in quantifying protein expression levels. Furthermore, PD-L1 expression, which is used as a companion diagnostic marker to determine indications for ICI treatment, is not a definitive biomarker. Thus, there is a need to identify superior biomarkers for predicting the efficacy of ICIs. In this study, we compared the correlation between conventional IHC-DAB and a novel PID method for detecting PD-L1 expression in patients with cancer treated with several ICIs. We analyzed whether the evaluation of PD-L1 protein expression using the PID method predicted the therapeutic efficacy of ICIs more reliably than the conventional DAB system.

## 2 Materials and methods

### 2.1 Ethics statement

The study was conducted in accordance with the guidelines of the Declaration of Helsinki and approved by the Ethics Committees of Showa University School of Medicine (approval number: 2772), Fukushima Medical University (approval number: 2019-262), Saitama Medical University (approval number: 2409), and Gunma University (approval number: HS2020-201). Informed consent was obtained from all patients involved in the study.

### 2.2 Patient selection

This study enrolled 155 patients with metastatic or recurrent cancer who were treated with ICIs. The patient cohort included patients with several types of cancer, including non-small cell lung carcinoma (NSCLC), gastric cancer, urothelial carcinoma, head and neck carcinoma, and malignant melanoma. This was a multicenter retrospective cohort study, and patients were diagnosed and treated at Showa University Hospital, Fukushima Medical University Hospital, Saitama Medical Center, and Gunma University Hospital from December 2015 to December 2022. All patients were treated with treatment regimens, including ICIs shown in **Table 3**, that were administered according to the clinical settings.

**Table 3:** Clinicopathological characteristics of all patients.

Characteristic	
Age (y) (mean ± SD)	67.5 ± 9.4
Sex (n)	
Male	119
Female	36
Cancer type (n)	
Non-small cell lung carcinoma	109
Gastric cancer (adenocarcinoma)	28
Urothelial carcinoma	11
Head and neck cancer (squamous carcinoma)	4
Malignant melanoma	3
Site of pathological specimen (n)	
Primary tumor	129
Metastatic tumor	26
ICI Regimen (n)	
Nivolumab monotherapy	101
Pembrolizumab monotherapy	45
Pembrolizumab + platinum-based chemotherapy	4
Atezolizumab + platinum-based chemotherapy	3
Nivolumab + ipilimumab	1
Atezolizumab monotherapy	1
PD-L1 PID score (mean (min - max))	2043 (556-15757)
PD-L1 expression (IHC) (n)	
≥50%	27
1-49%	59
<1%	60
Not evaluable	9

SD, standard deviation; ICI, immune checkpoint inhibitor; PID, phosphor-integrated dots; IHC, immunohistochemistry.

### 2.3 Assessment of the treatment response

Each patient’s treatment response was evaluated using computed tomography scans as imaging assessments. The treatment efficacy was evaluated according to the Response Evaluation Criteria in Solid Tumors version 1.1 (25). Overall survival (OS) was defined as the date from the start of the first administration of treatment to the date of mortality due to any cause or the last follow-up. Progression-free survival (PFS) was defined as the date from the start of treatment to the first documented progressive disease, mortality due to any cause, or the last follow-up, whichever occurred first. The cut-off date of follow-up was set as December 2022.

The “median PFS” or “median OS,” based on the results obtained from the phase III pivotal clinical trials (**Table 1**), were used to uniformly evaluate the patient treatment efficacies of patient populations with different types of cancer. The patient population was divided into two groups (responder and non-responder) or three groups (long responder, responder, and non-responder), according to the treatment response prescribed above for each cancer type and treatment regimen. We then performed an analysis to compare PD-L1 expression evaluated by the PID method in each group.

### 2.4 Evaluation of PD-L1 expression using the IHC-DAB method

All tumor tissue specimens evaluated for PD-L1 expression were obtained before each patient received ICI treatment. The staining procedure for IHC using DAB and the evaluation method for PD-L1 expression were performed according to clinical routines, which have already been used for companion diagnosis when ICIs are administered to patients with cancer. We prepared formalin-fixed, paraffin-embedded tissue samples obtained by biopsy or resection. To evaluate their PD-L1 IHC assay, 155 slides were tested using Dako PD-L1 IHC 28-8 PharmDX kits (anti-PD-L1 28-8 rabbit monoclonal primary antibody; Dako, Glostrup, Denmark) for nivolumab, PD-L1 IHC 22C3 PharmDX kits (anti-PD-L1 22C3 mouse monoclonal primary antibody; Agilent Technologies, Santa Clara, CA, USA) for pembrolizumab, and Ventana PD-L1 SP142 (anti-PD-L1 28-8 rabbit monoclonal primary antibody; Ventana, Antwerp, Belgium) for atezolizumab, according to the manufacturers’ instructions. Two independent pathologists were experts in interpreting the clinical cut-off values of the assays used in this study and independently evaluated all 155 immunostained slides. IHC tests were scored by pathologists in accordance with a previous article (26). Missing or damaged tissue cores were excluded from the analysis, as was the case with <100 total tumor cells for scoring. The 28-8, 22C3 assays were used to evaluate PD-L1 expression in tumor cells, whereas the SP142 assay was used to assess PD-L1 expression in both tumor and immune cells (27). Two methods were used to evaluate PD-L1 expression. The Tumor Proportion Score was evaluated as the percentage of PD-L1-positive cells among the total tumor cells, and it is used as a companion diagnostic tool for lung cancer. The Combined Positive Score was evaluated as the ratio of the number of PD-L1-positive tumor cells plus tumor-infiltrating immune cells, e.g., lymphocytes and macrophages, to the total number of tumor cells, and it is used to evaluate PD-L1 expression in other types of cancer (26).

### 2.5 Evaluation of PD-L1 expression with the fluorescence properties of PIDs

We used the same tumor tissue specimens to evaluate PD-L1 expression as for the IHC-DAB method. Tissues collected before the patient received ICI treatment were used for analysis. The quantitative immunohistochemical detection of proteins using PID nanoparticles has been previously described (21). The pathological sections were incubated with a primary antibody against PD-L1 22C3 (Agilent Technologies, Santa Clara, CA, USA). The sections were incubated with the secondary antibody, which is Universal Secondary Antibody (Ventana, Antwerp, Belgium), for 30 minutes at 25°C. Envision Flex Target Retrieval Solution was activated at a low pH for 20 minutes at 95°C. The sections were then treated with PID-conjugated streptavidin (0.06 nM) for 2 hours at 25°C. The negative control was prepared using PID staining but without the primary antibody. Hematoxylin was used for nuclear counterstaining. The sections were irradiated at 580 nm, and the fluorescence intensity was measured using a whole slide scanner (NanoZoomer S60; Hamamatsu Photonics K. K., Shizuoka, Japan) and a CMOS camera (ORCA-Flash version 4.0 LTPlus; Hamamatsu Photonics K. K., Shizuoka, Japan). Image capture, autofocusing, and shading correction were automated using the NDP.scan software (version 3.2.17, Hamamatsu Photonics K. K., Shizuoka, Japan) (**Figure 1B**). The number of PID particles was quantified using an automated exclusive QUIK software (version 1.0.1.0, Konica Minolta, Inc., Tokyo, Japan) in whole regions of the tumor tissue specimen. The input fluorescence images underwent high-pass filtering to eliminate background autofluorescence and noise. Subsequently, the positive bright spots resulting from the PIDs were accurately detected within fluorescence microscopy images. A previous article delved into examining the relationship between fluorescence intensity and particle count within a bright spot (21). Gonda et al. established a standard curve exhibiting a positive correlation between fluorescence signals and PID particle count. Employing this method, the fluorescence intensity of each positive bright spot analyzed in this study was translated into the corresponding PID particle count. The quantity of particles per 12 µm × 12 µm square area was visualized as a heat map. The “PD-L1 PID score” for each case was derived using the subsequent formula, computed as the mean value of the number of PID particles per 12 µm × 12 µm square area within each tissue specimen (**Figure 1C**). The unit of PID score is expressed as/144 µm^2^.


$$PID\;score\;\left(/144\;\mu m^{2}\right)=\frac{Sum\;of\;number\;of\;PID\;particles\;in\;whole\;regions\;of\;the\;specimen}{Number\;of\;square\;areas\;of\;12\;\mu m\times 12\;\mu m}$$

Therefore, the resulting fluorescent images were captured, processed, and homogenized using a computer image-processing method that quantified the number of PID nanoparticles.

### 2.6 Statistical analysis

Statistical tests were performed, and figures were created using GraphPad Prism 9.4.1 software (GraphPad Software Inc., San Diego, CA, USA). Student’s t-test and Fisher’s exact test were employed to compare the patient characteristics between the two groups. The Spearman correlation coefficient was used to analyze the associations between the variables. The comparison of PD-L1 expression values between the two groups was conducted using the Mann–Whitney U test. For multiple comparisons of PID scores between the three groups, statistical analyses were performed using one-way analysis of variance with the Dann–Bonferroni multiple comparison test. Statistical significance was defined at a p-value <0.05.

Regarding survival analyses, the survival durations (PFS and OS) of the patients were assessed using the Kaplan–Meier method and statistically analyzed using the log-rank tests. All tests were two-sided. When we compared between two groups using the log-rank tests, a p-value <0.05 was considered statistically significant. When performing comparisons among three groups with the Kaplan–Meier analysis, log-rank tests were performed for each of the triplicate pairs. P-values judged to be significantly different had to be adjusted and p-value <0.01667 (calculated 0.05 divided by 3) was determined to be statistically significant for comparison among three groups with Kaplan–Meier survival analysis.

## 3 Results

### 3.1 Clinicopathological characteristics

The clinicopathological characteristics of the patients are summarized in **Table 3**. Detailed patient information and data are presented in **Supplementary Table S1**. The median length of follow-up periods for all enrolled patients was 13.6 months (range, 0.5–69.1 months).

### 3.2 Correlation of PD-L1 expression between the IHC-DAB and PID methods

**Figure 2:**
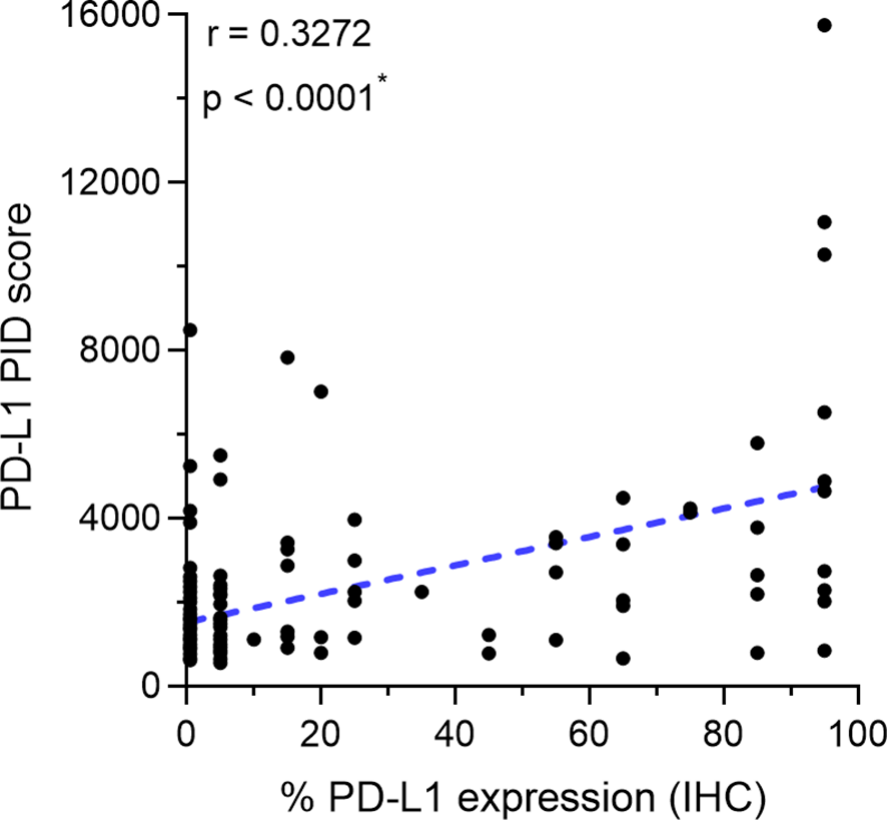
Correlation between programmed cell death ligand 1 (PD-L1) expression measured by immunohistochemical staining using 3,3´-diaminobenzidine chronogenesis (IHC-DAB) method and PD-L1 phosphor-integrated dot (PID) score. The Spearman correlation coefficient was used to analyze the correlation. A modest positive correlation is observed between PD-L1 expression measured by the IHC-DAB and PID methods (r=0.3272, p<0.0001). *Statistically significant: p<0.05.

**Figure 3:**
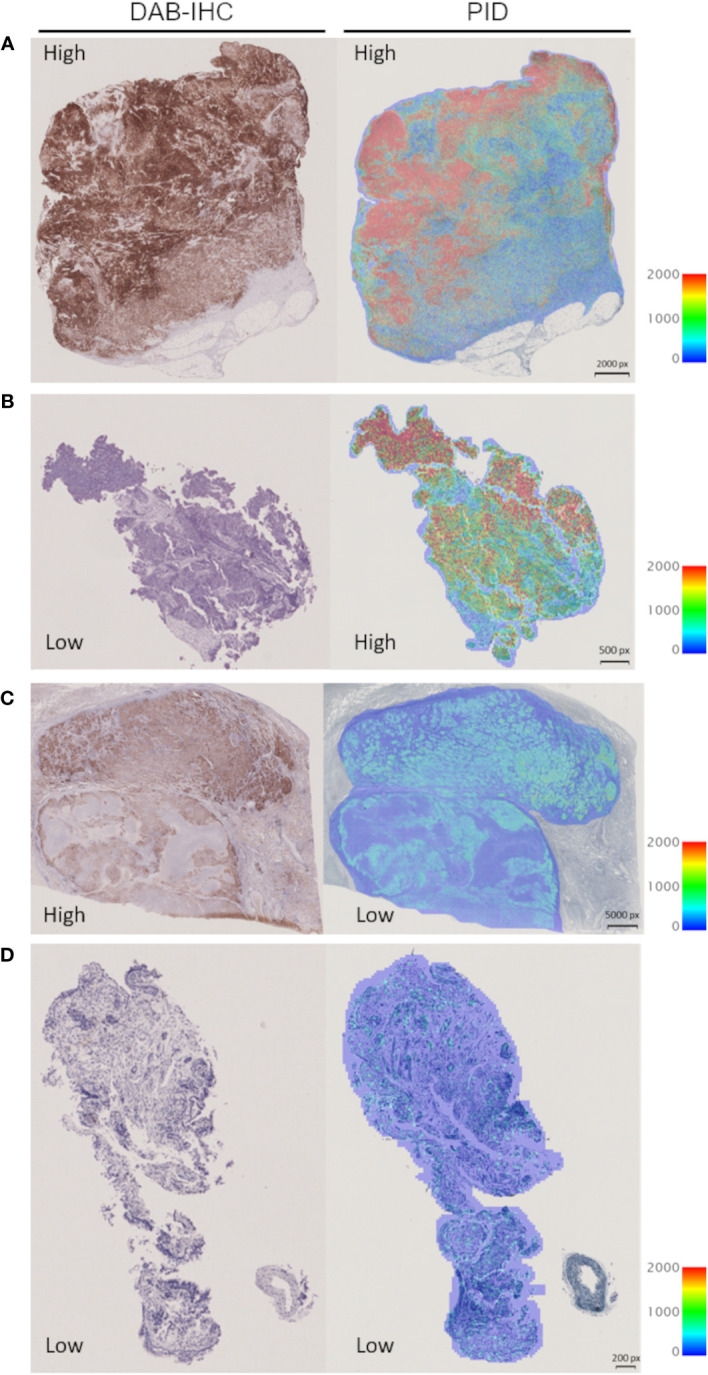
Representative images for visual comparison of programmed cell death ligand 1 (PD-L1) expression by the immunohistochemical staining using 3,3´-diaminobenzidine chronogenesis (IHC-DAB) and phosphor-integrated dot (PID) methods. **(A)** The case of high expression in IHC-DAB and high PID score: PD-L1 expression 90–100% (IHC-DAB), PD-L1 PID score 15757. **(B)** The case of low expression in IHC-DAB and high PID score: PD-L1 expression <1% (IHC-DAB), PD-L1 PID score 8487. **(C)** The case of high expression in IHC-DAB and low PID score: PD-L1 expression 90–100% (IHC-DAB), PD-L1 PID score 2024. **(D)** The case of low expression in IHC-DAB and low PID score: PD-L1 expression <1% (IHC-DAB), PD-L1 PID score 762. px, pixel.

We investigated the correlation between PD-L1 expression measured by the IHC-DAB method and PD-L1 expression analyzed by the PID method using the Spearman correlation coefficient test. Nine patients were excluded from the IHC-DAB test because of low tumor cell counts (<100 total tumor cells); therefore, 146 patients were included in the analysis. A modest positive correlation was observed between PD-L1 expression measured using the IHC-DAB and PID methods (r=0.3272, p<0.0001; **Figure 2**). In contrast, there were some cases in which PD-L1 expression levels were not positively correlated between the two methods, such as a low PD-L1 PID score, despite the high PD-L1 expression measured using the IHC-DAB method. We show several images comparing PD-L1 expression between the IHC-DAB and PID methods in **Figures 3A–D**. Several patients exhibited a high PD-L1 PID score, irrespective of the low PD-L1 expression level assessed by IHC-DAB. We identified 7 patients with PD-L1 (IHC-DAB) levels below 20% yet possessing a high PD-L1 PID score (>4000). We conducted a comparative analysis between this patient subgroup and the remaining patients to assess background characteristics. The examination revealed no statistically significant differences in patient background characteristics between the two patient groups (**Supplementary Table S2**).

### 3.3 Correlation between the PD-L1 PID score and patient survival

**Figure 4:**
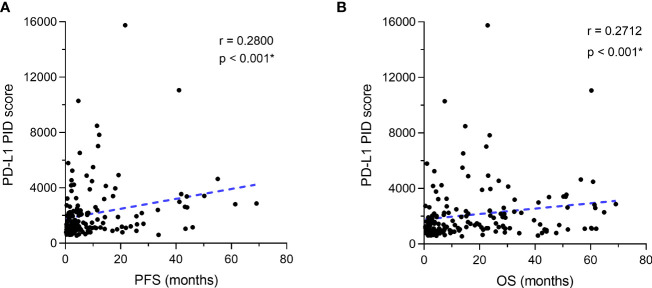
Correlation between programmed cell death ligand 1 (PD-L1) expression as the phosphor-integrated dot (PID) score and progression-free survival (PFS) and overall survival (OS). The Spearman correlation coefficient was used to analyze the correlation between the PD-L1 PID score and survival durations of **(A)** PFS and **(B)** OS. **(A)** There are weak positive correlations between the PID score and PFS in the overall cohort of patients. Similar to PFS, **(B)** a weak positive correlation with the PID score is observed for OS in the overall cohort. *Statistically significant: p<0.05.

The correlation between the PD-L1 PID score and survival duration (PFS and OS) was analyzed using the Spearman correlation coefficient test (n=155). There were weak positive correlations between the PID score and PFS in the overall cohort of patients (r=0.2800, p<0.001, **Figure 4A**). Similar to PFS, a weak positive correlation with the PID score was observed for OS in the overall cohort (r=0.2712, p<0.001, **Figure 4B**). PD-L1 PID scores before ICI treatment, as determined by the PID method, correlated with prolonged PFS and OS in patients with cancer who received ICI treatment.

### 3.4 Comparison of PD-L1 PID scores by the treatment efficacy of patients

**Figure 5:**
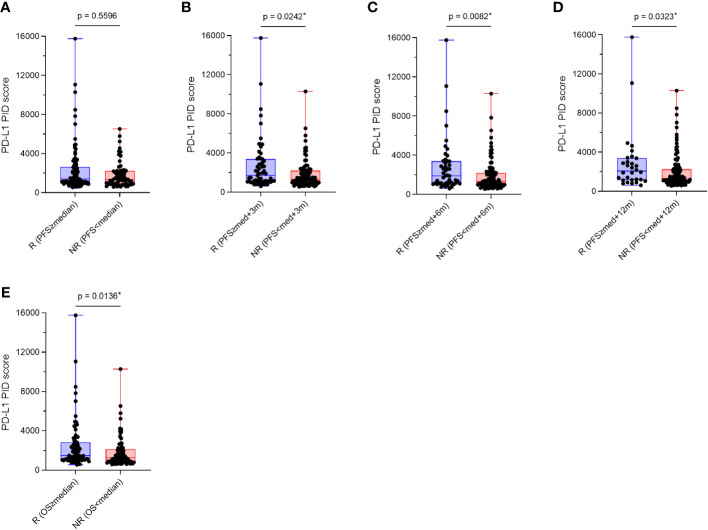
Comparison of programmed cell death ligand 1 (PD-L1) phosphor-integrated dot (PID) scores by treatment efficacy, responders and non-responders. Overall patient populations were divided into two groups, responders and non-responders, based on their treatment responses of immune checkpoint inhibitors, and PD-L1 PID scores were statistically compared between both groups. **(A)** PD-L1 PID scores are not significantly different in the analysis that distinguished non-responders from responders according to the “median PFS”. **(B–D)** However, PD-L1 PID scores are significantly higher in responders than in non-responders in this analysis for each patient’s PFS: **(B)** ≥”median PFS”+3 months, **(C)** ≥”median PFS”+6 months, and **(D)** ≥”median PFS”+12 months were defined as the responders. **(E)** Regarding OS, PD-L1 PID scores were significantly higher in R than in NR according to prolonged OS. R, responders; NR, non-responders; med, median. *Statistically significant: p<0.05.

We verified whether PD-L1 expression levels obtained using the PID method before treatment initiation predicted the efficacy of ICI treatment in patients. The overall patient population was divided into two groups, responder and non-responder, based on their treatment response to ICIs, and PD-L1 PID scores were statistically compared between the two groups using Mann–Whitney U test (n=155). The duration of PFS for the responder group was defined by four criteria: PFS of each patient was 1) longer than “median PFS,” 2) “median PFS”+3 months, 3) “median PFS”+6 months, 4) “median PFS”+12 months, based on “median PFS” data obtained from previous reported phase III pivotal trials evaluating ICI treatments (**Table 1**) (6–14). PD-L1 PID scores were not significantly different in the analysis that distinguished non-responders from responders according to the “median PFS” described above (p=0.5596, **Figure 5A**). However, PD-L1 PID scores were significantly higher in responders than in non-responders in this analysis for each patient’s PFS: ≥”median PFS”+3 months, ≥”median PFS”+6 months, and ≥”median PFS”+12 months were defined as responders (p=0.0242, **Figure 5B**; p=0.0082, **Figure 5C**; and p=0.0323, **Figure 5D**, respectively). Regarding OS, the duration of OS for the responders was defined by the criteria in which each patient’s OS was longer than the “median OS” reported in the previous pivotal trials (**Table 1**) (6–14). PD-L1 PID scores were significantly higher in responders than in non-responders according to prolonged OS (p=0.0136, **Figure 5E**).

**Figure 6:**
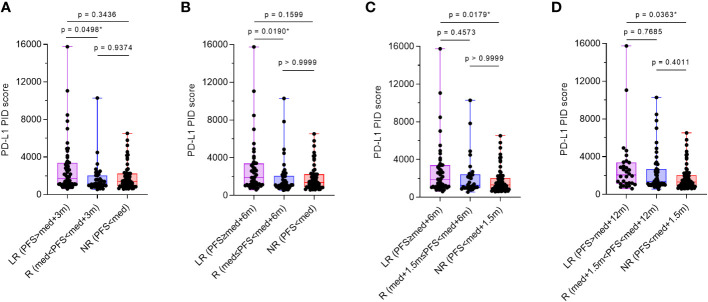
Comparison of programmed cell death ligand 1 (PD-L1) phosphor-integrated dot (PID) scores by treatment efficacy. The patient population was divided into three groups: long-responders, responders, and non-responders. One-way analysis of variance with the Dann–Bonferroni multiple comparison tests were performed to compare the three groups. **(A, B)** PID scores are significantly higher in long-responders than in responders (p=0.0292, **A**; p=0.0190, **B**) and **(C, D)** non-responders (p=0.0179, **C**; p=0.0363, **D**). LR, long-responders; R, responders; NR, non-responders; med, median. *Statistically significant: p<0.05.

Additionally, the patient population was divided into three groups: long responders, responders, and non-responders. PD-L1 expression as the PID score in each group was compared between the three groups with the “median PFS” reported from the pivotal trial as previously described (**Table 1**) (6–14). Multiple comparison test results were statistically analyzed using the Dann–Bonferroni multiple comparison test (n=155), and the PID scores were significantly higher in long responders than in responders (p=0.0498, **Figure 6A**; p=0.0190, **Figure 6B**), or non-responders (p=0.0179, **Figure 6C**; p=0.0363, **Figure 6D**). Based on these analyses of comparison between two and three groups, pre-ICI treatment PD-L1 expression measured as PID score by the PID method was associated with favorable PFS and OS in patients with cancer who received cancer immunotherapy with ICIs. The results regarding PFS suggest that PD-L1 PID scores might be predictive of better prognosis, as PID scores were higher in responders with longer PFS.

### 3.5 Kaplan–Meier survival analysis according to PD-L1 expression by the IHC-DAB method

**Figure 7:**
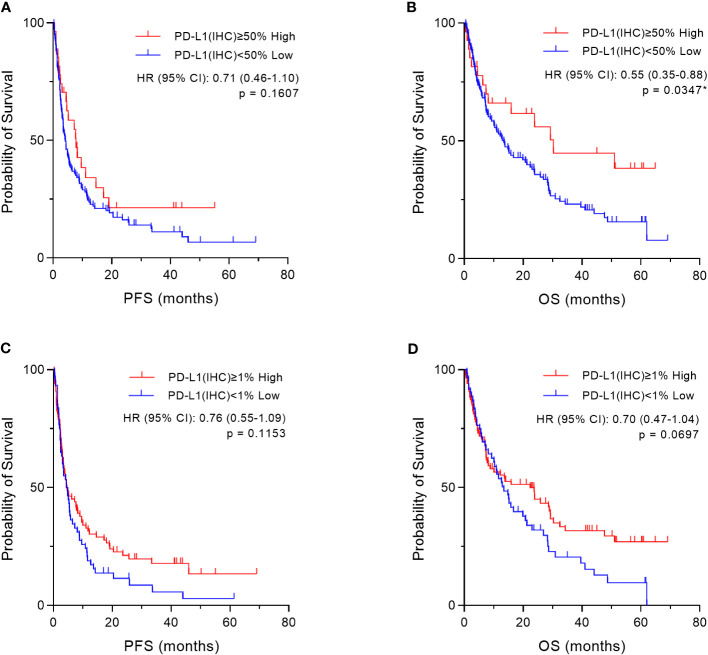
Kaplan–Meier survival analysis according to programmed cell death ligand 1 (PD-L1) expression by the immunohistochemical staining using 3,3´-diaminobenzidine chronogenesis (IHC-DAB) method in two groups. Based on the cut-off values (50% and 1%) of PD-L1 expression by the IHC-DAB method, the patient cohort was divided into two groups, “High” and “Low” according to PD-L1 expression levels by conventional IHC-DAB. In the overall patient population (n=155), we compared both groups using Kaplan–Meier survival analyses with log-rank tests for PFS and OS. **(A, C)** Regarding PFS, there are also no significant differences between the “High” and “Low” groups of PD-L1 expression by the IHC-DAB method, which were defined by the cut-offs of **(A)** 50% and **(C)** 1%. **(B)** OS is significantly prolonged in the “High” (PD-L1(IHC) ≥50%) group, **(D)** and there is a similar trend with a cut-off value of 1%, which is not statistically significant. HR, hazard ratio; CI, confidence interval. *Statistically significant: p<0.05.

Based on the cut-off values (50% and 1%) of PD-L1 expression by the IHC-DAB method, which is clinically applied (26), the patient cohort was divided into two groups, “high” and “low” according to PD-L1 expression levels by the conventional IHC-DAB method. Then, we compared both groups using Kaplan–Meier survival analyses with the log-rank tests for PFS and OS. In the overall patient population (n=155), OS was significantly prolonged in the PD-L1 high (PD-L1(IHC) ≥50%) group (p=0.0347, **Figure 7B**), and a similar trend was observed with a cut-off value of 1%, which was not statistically significant (p=0.0697, **Figure 7D**). Regarding PFS, there were no significant differences between the high and low groups of PD-L1 expression by the IHC-DAB method with cut-off values of 50% (p=0.1607, **Figure 7A**) and 1% (p=0.1153, **Figure 7C**). We additionally performed sub-analyses for the NSCLC patient cohort because of the large number of patients (n=109), but the results were not statistically significant (**Supplementary Figures 1A–D**).

**Figure 8:**
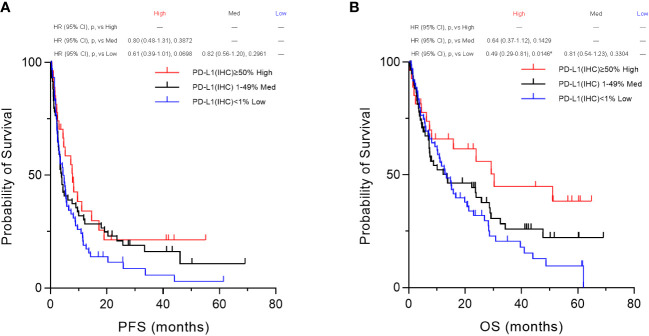
Kaplan–Meier survival analysis according to programmed cell death ligand 1 (PD-L1) expression by the immunohistochemical staining using 3,3´-diaminobenzidine chronogenesis (IHC-DAB) method between the three groups. Based on the PD-L1 cut-off values (50%, 1–49%, and 1%) evaluated by the IHC-DAB method, the patient population was divided into three groups of PD-L1 expression levels, “High,” “Medium,” and “Low” groups, and then we compared the three groups by performing Kaplan–Meier survival analyses with the log-rank tests for PFS and OS. **(A)** In the overall patient population (n=155), no significant results were obtained for PFS. **(B)** OS in the PD-L1 “High” (PD-L1(IHC) ≥50%) group was statistically prolonged compared to that of the PD-L1 “Low” (PD-L1(IHC) <1%) group (p=0.0146, **B**). HR, hazard ratio; CI, confidence interval; p, p-value; Med, medium. *Statistically significant: p<0.01667.

Furthermore, based on the PD-L1 cut-off values (50%, 1–49%, and 1%) evaluated by the IHC-DAB method, the patient population was divided into three groups according to PD-L1 expression levels, “High,” “Medium,” and “Low” groups, and then we compared the three groups using Kaplan–Meier survival analyses with the log-rank tests for PFS and OS. In the overall patient population (n=155), OS in the “High” (PD-L1(IHC) ≥50%) group was statistically prolonged compared to that of the “Low” (PD-L1(IHC) <1%) group (p=0.0146, **Figure 8B**). However, no significant results were obtained for PFS (**Figure 8A**) when the three groups were categorized based on PD-L1 expression by the IHC-DAB method. Sub-analyses for the NSCLC patient cohort were also performed (n=109), and there were no statistically significant findings in the Kaplan–Meier survival analyses for both PFS (**Supplementary Figure 2A**) and OS (**Supplementary Figure 2B**). Therefore, these analyses indicated that the PD-L1 expression levels defined by the conventionally used IHC-DAB method with PD-L1 cut-off values were not associated with favorable PFS and OS, except for the “High” (PD-L1(IHC) ≥50%) group in OS.

### 3.6 Determining the cut-off value of the PD-L1 PID score

There are no criteria for defining high or low PD-L1 expression using the proportional integral derivative method. To determine an appropriate cut-off value for the PD-L1 PID score, we defined “high” and “low” PD-L1 expression by the IHC-DAB method as the outcomes and plotted receiver operating characteristic (ROC) curves regarding the PID scores. The PID score with the highest value, calculated by the formula [Sensitivity - (1 + Specificity)], was defined as the most appropriate cut-off value by the Youden index to distinguish between high and low PD-L1 expression groups (28). Appropriate ROC curves with statistical significance were obtained when PD-L1 IHC-DAB cut-off values of 50%, 20%, and 10% were applied, and the most appropriate cut-off value of the PD-L1 PID score was 1863 (**Supplementary Figures 3A–H**).

Moreover, we divided the PID scores into three groups for analysis, as was done for the IHC-DAB method. The cut-off values for dividing the patients into three groups were determined using percentile values: 1) PID score ≥2359 (75th percentile) for the “High” group, 2) 948 (25th percentile)<PID score<2359 (75th percentile) for the “Medium” group, 3) PID score <948 (25th percentile) for the “Low” group (**Supplementary Figure 4**).

### 3.7 Kaplan–Meier survival analysis according to the PD-L1 PID score

**Figure 9:**
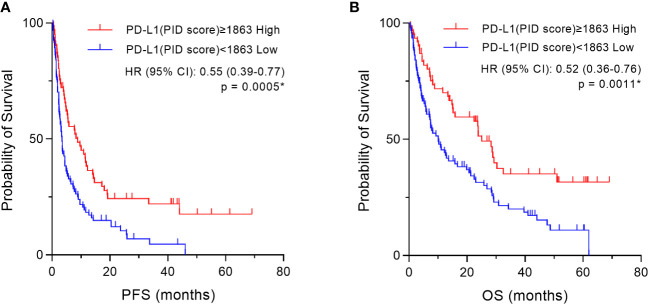
Kaplan–Meier survival analysis according to the programmed cell death ligand 1 (PD-L1) phosphor-integrated dot (PID) score in two groups. Based on the cut-off value (1863) of the PD-L1 PID score, the patient cohort was divided into two groups, “High” and “Low” according to PD-L1 expression levels by the PID method, and then we compared the two groups using Kaplan–Meier survival analyses with the log-rank tests for PFS and OS. In the overall patient population (n=155), both **(A)** PFS and **(B)** OS are prolonged in the “High” PD-L1 PID score group with high statistical significance. HR, hazard ratio; CI, confidence interval. *Statistically significant: p<0.05.

Based on the cut-off value (1863) of the PD-L1 PID score that was obtained above, the patient cohort was divided into two groups, “High” and “Low” according to the PD-L1 expression levels by the PID method, and then we compared the two groups using Kaplan–Meier survival analyses with log-rank tests for PFS and OS. In the overall patient population (n=155), PFS and OS were significantly prolonged in the “High” PD-L1 PID score group (p=0.0005, **Figure 9A** and p=0.0011, **Figure 9B**, respectively). We further performed sub-analyses of the NSCLC patient cohort (n=109). PFS was significantly longer in the “High” PID score group than in the “Low” PID score group (p=0.0325, **Supplementary Figure 5A**), and a similar trend was observed for OS in the NSCLC patient cohort (p=0.0575, **Supplementary Figure 5B**).

**Figure 10:**
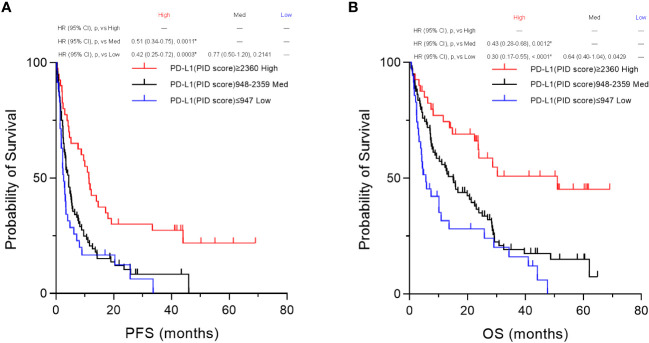
Kaplan–Meier survival analysis according to the programmed cell death ligand 1 (PD-L1) phosphor-integrated dot (PID) score between the three groups. Based on the 25th and 75th percentile values, PID scores were also divided into three groups for the survival analysis. We compared the three groups by performing Kaplan–Meier survival analyses with the log-rank tests for PFS and OS. In the overall patient population (n=155), both **(A)** PFS and **(B)** OS are significantly prolonged in the “High” PD-L1 PID score group compared with the “Medium” (PFS, p=0.0011, **A**; OS, p=0.0012, **B**) and “Low” PD-L1 PID score groups (PFS, p=0.0003, **A**; OS, p<0.0001, **B**). HR, hazard ratio; CI, confidence interval; p, p-value; Med, medium. *Statistically significant: p<0.01667.

Based on the percentile values, the PID scores were divided into three groups, “High,” “Med,” and “Low” groups, for survival analysis. Then, we compared the three groups using Kaplan–Meier survival analyses with the log-rank tests for PFS and OS. Only in these analyses of comparison among the three groups, a p-value <0.01667 was considered to be statistically significant. In the overall patient population (n=155), PFS was significantly prolonged in the “High” PD-L1 PID score group compared with the “Medium” (p=0.0011, **Figure 10A**) and “Low” PD-L1 PID score groups (p=0.0003, **Figure 10A**). Similar to PFS, the “High” PD-L1 PID score group had more favorable OS than the “Medium” (p=0.0012, **Figure 10B**) and “Low” PD-L1 PID score groups (p<0.0001, **Figure 10B**). In the NSCLC cohort (n=109), PFS and OS were longer in the “High” PD-L1 PID score group with strong statistical significance than in the “Medium” (p=0.0098, **Supplementary Figure 6A**; and p=0.0070, **Supplementary Figure 6B**, respectively) and “Low” PD-L1 PID score groups (p=0.0059, **Supplementary Figure 6A**; and p=0.0023, **Supplementary Figure 6B**, respectively). Therefore, the results demonstrated that when the PID score was used as the cut-off value for the PD-L1 expression level, the PID score more clearly predicted the treatment efficacy and prognosis of patients treated with ICIs.

## 4 Discussion

To evaluate whether quantitative detection of PD-L1 expression predicts the clinical outcomes of patients with cancer treated with ICIs, we demonstrated the expression of PD-L1 protein using two different immunohistochemical detection methods, the conventional IHC-DAB and PID system. From the results obtained herein, the quantitative evaluation of PD-L1 expression by the PID score appears to be more effective than the cut-off of PD-L1 expression by the IHC-DAB method in predicting the treatment efficacy and prognosis of patients with cancer treated with ICIs. PID scoring as a quantitative detection system is expected to resolve some limitations of the IHC-DAB method for quantifying protein expression levels.

Since the PID method was first reported in 2017 (21), researchers have focused on this technology and its practical applications. Gonda et al. published foundational articles on the PID system and established a novel method for quantitative protein evaluation by IHC using new fluorescent nanoparticles, called PIDs, with high sensitivity and a wide dynamic range (21). The PID method is strongly correlated with conventional human epidermal growth factor receptor 2 (HER2) testing methods using IHC-DAB (21, 29). In the present study, PD-L1 expression assessed using the conventional IHC-DAB method was positively correlated with that assessed using the PID method. Additionally, protein expression assessed by the PID method has been reported to have a positive linear correlation with that obtained by other methodologies such as fluorescence activated cell sorting analysis (21, 29) or enzyme-linked immunosorbent assay (ELISA) (24). Thus, the reproducibility of the PID method was confirmed by comparison with the other methods. It has also been verified whether protein expression evaluated using the PID method can be used as a biomarker for predicting treatment efficacy. The number of HER2-positive PID particles in breast cancer tissue analyzed from pretreatment biopsies have been shown to predict the therapeutic efficacy of the anti-HER2 antibody (trastuzumab) (21). Guo et al. showed that a high ratio of extranuclear-to-nuclear estrogen receptor alpha (ERα) in patients with hormone receptor-positive and HER2-negative breast cancer indicates a decreased likelihood of benefiting from hormone therapy (30). Similar to our study, the PID score for PD-L1 expression showed a higher prognostic value than protein detection using IHC-DAB (23). Quantitative evaluation of MYC protein expression using the PID method stratified OS in patients with diffuse large B-cell lymphoma more precisely than the conventional IHC-DAB method (31).

There have been limited studies on the quantitative evaluation of PD-L1 molecules using the PID method. In a previous study, PD-L1 expression in pancreatic ductal carcinoma was evaluated using IHC with PID, which could detect PD-L1 expression with higher sensitivity than conventional IHC-DAB. PD-L1 expression, evaluated using the PID method, predicts poor prognosis (23). Another study showed that digital immunostaining of PD-L1 expression was highly correlated with protein expression measured by other methods, such as ELISA and quantitative messenger RNA data generated by the nCounter system (24). Both studies are valuable in that they evaluated PD-L1 expression using the novel PID method, but they did not validate whether it predicts the efficacy of ICI treatment. In our study, we not only compared the PID method with the conventional IHC-DAB method in assessing PD-L1 expression but also analyzed the relationship between PD-L1 expression by IHC-DAB and treatment response to ICIs using pre-ICI treatment tissue specimens from 155 patients with cancer. When the patients were classified into responder and non-responder groups based on the duration of PFS and OS, the PD-L1 PID scores in the responder group were higher than those in the non-responders. As our data showed that PID scores tended to be higher in patients with a longer PFS, it is possible that PID scores were better at predicting long responders, which is a hallmark of ICI treatment. Furthermore, when we performed survival analysis by dividing patients into high and low PD-L1 PID score groups, PFS and OS were significantly prolonged in patients with high PID scores. However, when the PD-L1 expression level was evaluated using the conventional DAB method, neither PFS nor OS was significant and could not predict treatment response or prognosis. We found that the PD-L1 expression level evaluated using the PID method has the potential to be a better biomarker than the IHC-DAB method. There are several possible reasons why the two analysis methods gave different results. The main limitation of the IHC-DAB method is the dependence of the staining intensity on the enzymatic activity of HRP, which in turn is influenced by factors such as temperature, reaction time, and HRP substrate concentration. Furthermore, the efficacy of the IHC-DAB method is curtailed by the subjective selection of noteworthy fields of view by pathologists and their subsequent visual assessment of PD-L1 expression, which prevents quantitative evaluation and lacks objectivity. Conversely, the PID method features brightness levels 100 times greater than conventional fluorescent nanoparticles, along with 10 times greater lightfastness compared to existing fluorescent dyes (21). These distinctive attributes equip the PID method with the capacity to assess protein expression assessments in a more quantitative and accurate manner than the DAB method. Additionally, the capability of the PID method to comprehensively analyze entire regions of tumor tissue specimens permits the evaluation of PD-L1 expression in whole areas that conventional visual inspection by pathologists may not fully capture. These factors likely contribute to the disparities in results observed between the DAB and PID methods.

Furthermore, the PID method has been applied to research other than the search for predictive biomarkers of therapeutic efficacy. Guo et al. performed PID analysis using the nearest neighbor method, which takes advantage of the ability to analyze the location of detected proteins in cells and tissues. ERα expression in nuclear and extranuclear regions was detected and quantitatively analyzed, resulting in higher sensitivity and specificity than conventional IHC-DAB in patients with breast cancer (30). Suzuki et al. applied PID imaging to study antibody drugs to elucidate their mechanism of action. They evaluated the intratumor pharmacokinetics using PID imaging analysis, which can assess the distribution of proteins to tumor target sites at the microlevel, to analyze the intratumor distribution of a novel HER2-targeted antibody drug conjugate, trastuzumab deruxtecan (32). PID imaging analysis is expected to be used not only to detect biomarkers such as HER2 and PD-L1 expressed in tumor tissue but also as an ideal tool for elucidating the mechanism of action of antibody drugs in tumor tissue in the clinical setting. Moreover, as Inamura et al. analyzed the expression of colony stimulating factor-1 receptor-expressing tumor-associated macrophages in lung cancer tumor tissue (33), PID imaging technology will be increasingly applied to analyze the immune microenvironment in tumor tissue.

We found no significant difference in PID scores between responders and non-responders when using the “median PFS” reported in the pivotal trial as the cut-off, but significant results were obtained when patients were divided by “median PFS”+3 months, “median PFS”+6 months, and “median PFS”+12 months. In clinical trials of ICIs, PFS can be attributed to tumor shrinkage (pseudo-progression) following disease progression (PD) or to longer survival after PD, both of which suggest a delayed effect of ICIs. Previous studies have reported that excluding modified PFS, which excludes early PD events up to 3 months after randomization, is a more accurate surrogate endpoint for OS than actual PFS (34). Thus, considering the early PD of approximately 3 months, it is possible that a median PFS of 3 months or more would be reasonable to obtain significant results.

The present study has several limitations. It is a retrospective analysis, and there lies the aspect that it solely served as an exploratory investigation into the utility of PD-L1 expression through the PID method. In terms of the study design, the enrolled patients exhibited heterogeneity and encompassed various cancer types. The inclusion of diverse cancer types in this study gives rise to discrepancies in the approach to evaluating PD-L1 expression by the IHC-DAB method between NSCLC and other cancer types. Our assessment of PD-L1 expression by the IHC-DAB method aligns with the method employed in clinical practice. TPS serves as a companion diagnostic tool for lung cancer, whereas CPS is utilized for assessing PD-L1 expression in other cancer types within clinical settings. Furthermore, the determination of the cut-off value of the PD-L1 PID score also remains a challenge. Currently, no recommended or established cut-off values exist for evaluating PD-L1 expression using the PID method. In this study, we established our own cut-off values utilizing ROC curves and percentile values. These cut-off values for PD-L1 PID scores may vary based on patient background, such as different cancer types. To resolve these issues and verify our results, conducting a prospective study with a homogenized patient population is imperative. We are in the process of planning a clinical trial to investigate PD-L1 expression through the PID method in the future.

## 5 Conclusions

We evaluated PD-L1 expression using highly sensitive quantitative immunohistochemistry with fluorescent nanoparticles (PIDs) in 155 patients with unresectable, recurrent, or metastatic cancer treated with ICIs, and compared it with that using the conventional IHC-DAB method. Evaluation of PD-L1 expression by the IHC-DAB and PID methods showed a positive correlation. The quantitative assessment of PD-L1 expression using the PID method predicted responders to ICI treatment. Furthermore, PFS and OS were significantly prolonged in the group with higher PD-L1 PID scores, suggesting that quantitative evaluation of PD-L1 expression by the PID method could be a biomarker for predicting treatment efficacy and patient prognosis of ICI treatment. It is significant that the PID method was able to identify the favorable prognosis group that could not be detected using conventional DAB staining. We propose prospective studies and further research on the quantitative evaluation of PD-L1 expression using the innovative PID method with the aim of adapting this methodology to clinical practice.

## Data availability statement

The raw data supporting the conclusions of this article will be made available by the authors, without undue reservation.

## Ethics statement

The studies involving humans were approved by the Ethics Committees of Showa University School of Medicine (approval number: 2772), Fukushima Medical University (approval number: 2019-262), Saitama Medical University (approval number: 2409), and Gunma University (approval number: HS2020-201). The studies were conducted in accordance with the local legislation and institutional requirements. The participants provided their written informed consent to participate in this study. Written informed consent was obtained from the individual(s) for the publication of any potentially identifiable images or data included in this article.

## Author contributions

RO: Conceptualization, Data curation, Formal Analysis, Investigation, Methodology, Resources, Validation, Visualization, Writing – original draft, Software. SMi: Investigation, Methodology, Resources, Validation, Writing – review & editing. SMu: Investigation, Resources, Writing – review & editing. YT: Investigation, Resources, Writing – review & editing. YF: Methodology, Resources, Writing – review & editing. KI: Methodology, Resources, Writing – review & editing. NOn: Methodology, Resources, Writing – review & editing. TS: Methodology, Resources, Writing – review & editing. MW: Methodology, Resources, Writing – review & editing. DT: Methodology, Resources, Writing – review & editing. TG: Methodology, Resources, Writing – review & editing. AH: Investigation, Resources, Validation, Writing – review & editing. KH: Investigation, Resources, Validation, Writing – review & editing. HA: Investigation, Resources, Validation, Writing – review & editing. MS: Investigation, Resources, Validation, Writing – review & editing. YH: Investigation, Resources, Validation, Writing – review & editing. TI: Investigation, Resources, Validation, Writing – review & editing. RS: Investigation, Resources, Validation, Writing – review & editing. NI: Investigation, Resources, Validation, Writing – review & editing. TTsur: Validation, Writing – review & editing, Investigation, Resources. EM: Investigation, Resources, Writing – review & editing, Validation. ST: Writing – review & editing, Supervision, Validation. KN: Investigation, Resources, Writing – review & editing. NOk: Investigation, Resources, Writing – review & editing. KY: Supervision, Validation, Writing – review & editing, Investigation, Resources. MT: Supervision, Writing – review & editing, Validation. YK: Supervision, Writing – review & editing, Validation. TYaj: Investigation, Resources, Supervision, Writing – review & editing. HI: Supervision, Writing – review & editing, Investigation, Resources. HS: Supervision, Writing – review & editing, Investigation, Resources. TYam: Investigation, Resources, Validation, Writing – review & editing, Methodology. SK: Supervision, Validation, Writing – review & editing. TTsun: Conceptualization, Investigation, Project administration, Resources, Supervision, Validation, Writing – review & editing, Funding acquisition. SW: Conceptualization, Data curation, Investigation, Methodology, Project administration, Resources, Supervision, Validation, Writing – review & editing, Funding acquisition.

## References

[1] Taube JM Klein A Brahmer JR Xu H Pan X Kim JH. Association of PD-1, PD-1 ligands, and other features of the tumor immune microenvironment with response to anti-PD-1 therapy. Clin Cancer Res (2014) 20:5064–74. doi: 10.1158/1078-0432.CCR-13-3271 PMC4185001 24714771

[2] Michot JM Bigenwald C Champiat S Collins M Carbonnel F Postel-Vinay S. Immune-related adverse events with immune checkpoint blockade: a comprehensive review. Eur J Cancer (2016) 54:139–48. doi: 10.1016/j.ejca.2015.11.016 26765102

[3] Le Mercier I Lines JL Noelle RJ. Beyond CTLA-4 and PD-1, the generation Z of negative checkpoint regulators. Front Immunol (2015) 6:418. doi: 10.3389/fimmu.2015.00418 26347741 PMC4544156

[4] Chen L Flies DB. Molecular mechanisms of T cell co-stimulation and co-inhibition. Nat Rev Immunol (2013) 13:227–42. doi: 10.1038/nri3405 PMC3786574 23470321

[5] Vaddepally RK Kharel P Pandey R Garje R Chandra AB. Review of indications of FDA-approved immune checkpoint inhibitors per NCCN guidelines with the level of evidence. Cancers (Basel) (2020) 12:738. doi: 10.3390/cancers12030738 32245016 PMC7140028

[6] Reck M Rodríguez-Abreu D Robinson AG Hui R Csőszi T Fülöp A. Five-year outcomes with pembrolizumab versus chemotherapy for metastatic non-small-cell lung cancer with PD-L1 tumor proportion score ≥ 50. J Clin Oncol (2021) 39:2339–49. doi: 10.1200/JCO.21.00174 PMC8280089 33872070

[7] Borghaei H Gettinger S Vokes EE Chow LQM Burgio MA de Castro Carpeno J. Five-year outcomes from the randomized, phase III trials checkMate 017 and 057: nivolumab versus docetaxel in previously treated non-small-cell lung cancer. J Clin Oncol (2021) 39:723–33. doi: 10.1200/JCO.20.01605 PMC8078445 33449799

[8] Rittmeyer A Barlesi F Waterkamp D Park K Ciardiello F von Pawel J. Atezolizumab versus docetaxel in patients with previously treated non-small-cell lung cancer (OAK): a phase 3, open-label, multicentre randomised controlled trial. Lancet (2017) 389:255–65. doi: 10.1016/S0140-6736(16)32517-X PMC6886121 27979383

[9] Boku N Satoh T Ryu MH Chao Y Kato K Chung HC. Nivolumab in previously treated advanced gastric cancer (ATTRACTION-2): 3-year update and outcome of treatment beyond progression with nivolumab. Gastric Cancer (2021) 24:946–58. doi: 10.1007/s10120-021-01173-w PMC8205916 33743112

[10] Fradet Y Bellmunt J Vaughn DJ Lee JL Fong L Vogelzang NJ. Randomized phase III KEYNOTE-045 trial of pembrolizumab versus paclitaxel, docetaxel, or vinflunine in recurrent advanced urothelial cancer: results of >2 years of follow-up. Ann Oncol (2019) 30:970–6. doi: 10.1093/annonc/mdz127 PMC6594457 31050707

[11] Gillison ML Blumenschein G Fayette J Guigay J Colevas AD Licitra L. Long-term outcomes with nivolumab as first-line treatment in recurrent or metastatic head and neck cancer: subgroup analysis of checkMate 141. Oncologist (2022) 27:e194–8. doi: 10.1093/oncolo/oyab036 PMC8895496 35641218

[12] Wolchok JD Chiarion-Sileni V Gonzalez R Grob JJ Rutkowski P Lao CD. Long-term outcomes with nivolumab plus ipilimumab or nivolumab alone versus ipilimumab in patients with advanced melanoma. J Clin Oncol (2022) 40:127–37. doi: 10.1200/JCO.21.02229 PMC8718224 34818112

[13] Rodríguez-Abreu D Powell SF Hochmair MJ Gadgeel S Esteban E Felip E. Pemetrexed plus platinum with or without pembrolizumab in patients with previously untreated metastatic nonsquamous NSCLC: protocol-specified final analysis from KEYNOTE-189. Ann Oncol (2021) 32:881–95. doi: 10.1016/j.annonc.2021.04.008 33894335

[14] West H McCleod M Hussein M Morabito A Rittmeyer A Conter HJ. Atezolizumab in combination with carboplatin plus nab-paclitaxel chemotherapy compared with chemotherapy alone as first-line treatment for metastatic non-squamous non-small-cell lung cancer (IMpower130): a multicentre, randomised, open-label, phase 3 trial. Lancet Oncol (2019) 20:924–37. doi: 10.1016/S1470-2045(19)30167-6 31122901

[15] Wada S Kobayashi S Tsunoda T. Future prospects for cancer immunotherapy - Strategies for ineffective cancers. Hum Vaccin Immunother (2022) 18:2031699. doi: 10.1080/21645515.2022.2031699 35077339 PMC8993051

[16] Hamada K Tsunoda T Yoshimura K. Emerging immune-monitoring system for immune checkpoint inhibitors. Life (Basel) (2022) 12:1229. doi: 10.3390/life12081229 36013407 PMC9410458

[17] Tsao MS Kerr KM Kockx M Beasley MB Borczuk AC Botling J. PD-L1 immunohistochemistry comparability study in real-life clinical samples: results of blueprint phase 2 project. J Thorac Oncol (2018) 13:1302–11. doi: 10.1016/j.jtho.2018.05.013 PMC8386299 29800747

[18] Borghaei H Paz-Ares L Horn L Spigel DR Steins M Ready NE. Nivolumab versus docetaxel in advanced nonsquamous non-small-cell lung cancer. N Engl J Med (2015) 373:1627–39. doi: 10.1056/NEJMoa1507643 PMC5705936 26412456

[19] Brahmer J Reckamp KL Baas P Crinò L Eberhardt WE Poddubskaya E. Nivolumab versus docetaxel in advanced squamous-cell non-small-cell lung cancer. N Engl J Med (2015) 373:123–35. doi: 10.1056/NEJMoa1504627 PMC4681400 26028407

[20] Thunnissen E Allen TC Adam J Aisner DL Beasley MB Borczuk AC. Immunohistochemistry of pulmonary biomarkers: A perspective from members of the pulmonary pathology society. Arch Pathol Lab Med (2018) 142:408–19. doi: 10.5858/arpa.2017-0106-SA 28686497

[21] Gonda K Watanabe M Tada H Miyashita M Takahashi-Aoyama Y Kamei T. Quantitative diagnostic imaging of cancer tissues by using phosphor-integrated dots with ultra-high brightness. Sci Rep (2017) 7:7509. doi: 10.1038/s41598-017-06534-z 28790306 PMC5548777

[22] Stack EC Wang C Roman KA Hoyt CC. Multiplexed immunohistochemistry, imaging, and quantitation: a review, with an assessment of Tyramide signal amplification, multispectral imaging and multiplex analysis. Methods (2014) 70:46–58. doi: 10.1016/j.ymeth.2014.08.016 25242720

[23] Yamaki S Yanagimoto H Tsuta K Ryota H Kon M. PD-L1 expression in pancreatic ductal adenocarcinoma is a poor prognostic factor in patients with high CD8+ tumor-infiltrating lymphocytes: highly sensitive detection using phosphor-integrated dot staining. Int J Clin Oncol (2017) 22:726–33. doi: 10.1007/s10147-017-1112-3 PMC5533855 28314962

[24] Fujisawa T Tsuta K Yanagimoto H Yagi M Suzuki K Nishikawa K. Quantitative immunohistochemical assay with novel digital immunostaining for comparisons of PD-L1 antibodies. Mol Clin Oncol (2019) 10:391–6. doi: 10.3892/mco.2019.1801 PMC6388504 30847180

[25] Eisenhauer EA Therasse P Bogaerts J Schwartz LH Sargent D Ford R. New response evaluation criteria in solid tumours: revised RECIST guideline (version 1.1). Eur J Cancer (2009) 45:228–47. doi: 10.1016/j.ejca.2008.10.026 19097774

[26] Paver EC Cooper WA Colebatch AJ Ferguson PM Hill SK Lum T. Programmed death ligand-1 (PD-L1) as a predictive marker for immunotherapy in solid tumours: a guide to immunohistochemistry implementation and interpretation. Pathology (2021) 53:141–56. doi: 10.1016/j.pathol.2020.10.007 33388161

[27] Krigsfeld GS Prince EA Pratt J Chizhevsky V William Ragheb J Novotny J. Analysis of real-world PD-L1 IHC 28-8 and 22C3 pharmDx assay utilisation, turnaround times and analytical concordance across multiple tumour types. J Clin Pathol (2020) 73:656–64. doi: 10.1136/jclinpath-2020-206466 PMC7513267 32591352

[28] Bantis LE Nakas CT Reiser B. Construction of confidence regions in the ROC space after the estimation of the optimal Youden index-based cut-off point. Biometrics (2014) 70:212–23. doi: 10.1111/biom.12107 24261514

[29] Hicks DG Buscaglia B Goda H McMahon L Natori T Turner B. A novel detection methodology for HER2 protein quantitation in formalin-fixed, paraffin embedded clinical samples using fluorescent nanoparticles: an analytical and clinical validation study. BMC Cancer (2018) 18:1266. doi: 10.1186/s12885-018-5172-1 30563489 PMC6299600

[30] Guo Z Tada H Kitamura N Hamada Y Miyashita M Harada-Shoji N. Automated quantification of extranuclear ERα using phosphor-integrated dots for predicting endocrine therapy resistance in HR+/HER2- breast cancer. Cancers (Basel) (2019) 11:526. doi: 10.3390/cancers11040526 31013810 PMC6520781

[31] Takayanagi N Momose S Kikuchi J Tanaka Y Anan T Yamashita T. Fluorescent nanoparticle-mediated semiquantitative MYC protein expression analysis in morphologically diffuse large B-cell lymphoma. Pathol Int (2021) 71:594–603. doi: 10.1111/pin.13131 34171161

[32] Suzuki M Yagishita S Sugihara K Ogitani Y Nishikawa T Ohuchi M. Visualization of intratumor pharmacokinetics of [fam-] trastuzumab deruxtecan (DS-8201a) in HER2 heterogeneous model using phosphor-integrated dots imaging analysis. Clin Cancer Res (2021) 27:3970–9. doi: 10.1158/1078-0432.CCR-21-0397 33980613

[33] Inamura K Shigematsu Y Ninomiya H Nakashima Y Kobayashi M Saito H. CSF1R-expressing tumor-associated macrophages, smoking and survival in lung adenocarcinoma: analyses using quantitative phosphor-integrated dot staining. Cancers (Basel) (2018) 10:252. doi: 10.3390/cancers10080252 30065206 PMC6115958

[34] Wang ZX Wu HX Xie L Lin WH Liang F Li J. Exploration of modified progression-free survival as a novel surrogate endpoint for overall survival in immuno-oncology trials. J Immunother Cancer (2021) 9:e002114. doi: 10.1136/jitc-2020-002114 33795385 PMC8021890

